# Changes in Diversity of Silica-Scaled Chrysophytes during Lake–River–Reservoir Transition (Baikal–Angara–Irkutsk Reservoir)

**DOI:** 10.3390/life13102052

**Published:** 2023-10-13

**Authors:** Anna Bessudova, Yuri Galachyants, Alena Firsova, Diana Hilkhanova, Maria Nalimova, Artyom Marchenkov, Ivan Mikhailov, Maria Sakirko, Yelena Likhoshway

**Affiliations:** Limnological Institute, Siberian Branch of the Russian Academy of Sciences, 3 Ulan-Batorskaya, 664033 Irkutsk, Russiadiana.khilkhanova@mail.ru (D.H.); nalimov.maria@yandex.ru (M.N.);

**Keywords:** river regulation, bioindicators, changes of diversity, distribution of silica-scaled chrysophytes, ecology

## Abstract

Hydroelectric dams create new ecosystems such as reservoirs. Several hydroelectric dams forming shallow reservoirs were built on the Angara River flowing out of Lake Baikal. The first of them in downstream Angara is Irkutsk Reservoir, with several shallow bays. Since silica-scaled chrysophytes are effective bioindicators for aquatic ecosystems, this paper aimed to determine their distribution, taxonomic structure and species richness in South Baikal and Irkutsk Reservoir, which have different environmental parameters. Thirty-one species were found using scanning and transmission electron microscopy. Only seven of them inhabited South Baikal in June 2023 at 3.66–4.51 °C and pH 7.80–8.24, with *Chrysosphaerella baicalensis*, *Spiniferomonas trioralis* f. *cuspidata* and *Mallomonas alpina* being prevalent. Only one species (*M. alpina*) was dominant in Irkutsk Reservoir at all stations at a water temperature of 5.33–11.55 °C and pH 8.10–8.52, alongside three other abundant species, *Synura* cf. *glabra*, *Mallomonas acaroides* and *M. crassisquama.* The maximum number of species (23) was found in a shallow bay of the reservoir at maximal values of temperature (11.5 °C) and pH (8.57) and minimal values of phosphate and nitrate concentrations during the study. The enrichment of Irkutsk Reservoir in species of silica-scaled chrysophytes was due both to cosmopolitan widespread and polyzonal species as well as to rare boreal, arctic–boreal, and unknown, possibly new species.

## 1. Introduction

Reservoirs emerged following the creation of hydroelectric dams and are located, as a rule, near human settlements. They are used for fishing and recreation and as drinking water supplies [1]. Thereby, monitoring water quality in reservoirs is a very important issue. Phytoplankton [1,2,3,4] are very well known to be one of the most effective indicators of aquatic system changes, since the species has a very fast response to any change in water environment, changing the taxonomic structure of its community and/or number of species in the community [1,3,5,6,7] due to their short life cycle and broad spatial distribution. Some Bacillariophyta and Cyanoprokaryota species are known as indicators of water quality [8,9,10]. Nevertheless, silica-scaled chrysophytes (Chrysophyceae Pascher) have not yet been well studied for that purpose, though they are known to be very sensitive to any environmental changes [11,12]. This limitation is induced by light microscopy usually applied by researchers analyzing phytoplankton, since it is not able to fully detect the species composition of silica-scaled chrysophytes. Only scanning and transmission electron microscopy (SEM and TEM) allow for the recognition of structural peculiarities of scales and the identification of species.

The diversity of silica-scaled chrysophytes in reservoirs may be quite high according to data of electron microscopy. E.g., 29 and 20 species [13] were detected in the large northern reservoirs of the Volga catchment area such as Sheksna and Rybinsk Reservoirs, respectively. Large reservoirs of the Angara catchment area such as Khantai and Boguchany Reservoirs showed the presence of 23 [14] and 23 [15] species, respectively. Eleven [16] species were detected in Kolyma Reservoir, while twenty-six [17] were found in a reservoir in Hungary. It is also known that silica-scaled chrysophytes occur in reservoirs of tropical areas [9,10,18], but they differ, as a rule, in species composition and have less diversity compared with that of reservoirs in the northern latitudes. Some species are part of a functional group of phytoplankton, reaching numbers that may be comparable with those of diatoms [10]. Silica-scaled chrysophytes may play an important role in plankton during the whole open water period [13,17] thanks to differentiation by nutritional preferences [19]. E.g., heterotrophic species of the genus *Paraphysomonas* De Saedeleer were prevalent during the ice period (March) in the upstream side of Boguchany Reservoir. In summer (July), when the bloom of large-celled diatoms was over, the most abundant were mixotrophic species of the *Spiniferomonas* Takahashi genus and autotrophic species of the *Mallomonas* Perty genus [15] along with Chlorophyta.

The feature of Irkutsk Reservoir is that it is the first in a cascade of reservoirs on the Angara River. It was created by a hydroelectric dam built 56 km downstream of Lake Baikal. It is a flowing water reservoir with a full turnover twice a month, which is greatly impacted by water from Lake Baikal. This is reflected in phytoplankton species diversity [20,21] and limits the development of Cyanoprokaryota typical of many reservoirs [4,22,23,24,25,26].

Such a dynamic lake–river–reservoir transition with gradients of water parameters makes it a unique model natural object for the detailed analysis of peculiarities of the taxonomic structure and distribution of species of silica-scaled chrysophytes previously found in Irkutsk Reservoir in a quantity of five species [20], and for the determination of a degree of continuity of species composition of these organisms during the transfer from the cold water of South Baikal to the warmer water of Irkutsk Reservoir and its bays. There is a necessity to obtain more data on this ecosystem; since Irkutsk Reservoir is located within Irkutsk, local people use it for recreation and it is used for urban water supply. The aim of this article is to identify the features of the distribution of silica-scaled chrysophytes, their taxonomic structure and species richness in South Baikal and Irkutsk Reservoir, which have different environmental parameters.

## 2. Materials and Methods

### 2.1. Study Site

**Figure 1:**
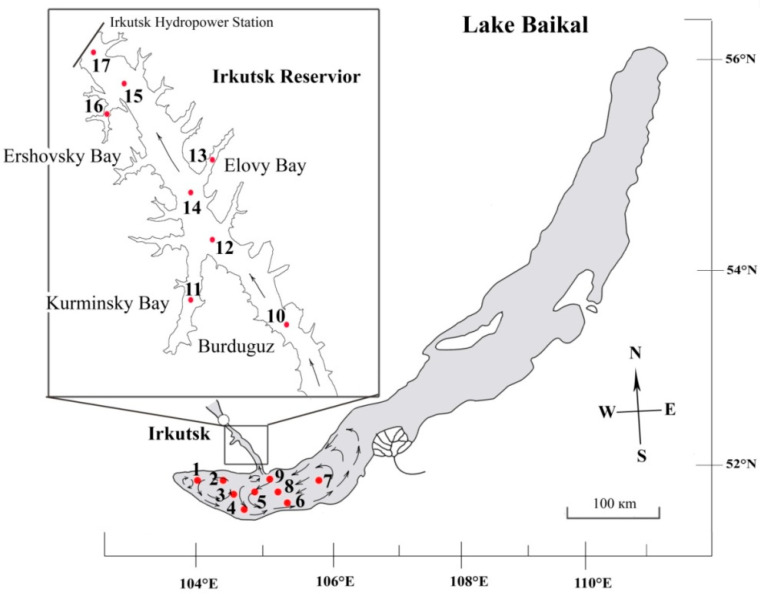
Sampling map of South Baikal and Irkutsk Reservoir (numbers indicate sampling stations, arrows—direction of currents [31]).

Lake Baikal is the deepest cold freshwater oligotrophic lake containing 20% of the planet’s fresh pure water [27]. The lake started emerging at the early beginning of the Miocene as a result of a rift filled with fresh water. The rift developed from south to north forming three basins (South, Central, and North) [28,29]. The basins differ in maximal depth [30] and horizontal currents [31]. In order to underline the differences, the basins are often named South, Central and North Baikal. The maximal depth in South Baikal reaches 1473 m, the horizontal currents go counterclockwise, and only in the very south do they move clockwise [31] (Figure 1).

The Angara River is the sole river flowing out of Lake Baikal. Its length up to the fall in to the Yenisei is 1779 km. A high vertical drop (380 m) from the source to the fall makes the Angara favorable for the creation of hydroelectric dams. A cascade of four hydroelectric dams was constructed on the river, resulting in four artificial lakes: Irkutsk, Bratsk, Ust-Ilimsk and Boguchany Reservoirs. The Angara never ices over at the outflow from Lake Baikal and below the dam within the boundaries of Irkutsk; its temperature in winter is 0.3–1.7 °C. The ice cover near the dam lasts an average of 145 days [20].

A low-head hydroelectric dam was constructed during 1950–1959 on the Angara River within Irkutsk and formed a reservoir of the same name. The maximal depth of the reservoir is 35 m; its surface water area is 154 km^2^ [32]. It is 1 km wide near the source and 2.5 km wide at the dam. Irkutsk Reservoir is an impoundment on-stream artificial lake with the prevalence of discharge currents [32].

### 2.2. Sampling and Field Work

Water sampling was carried out during 22–26 July onboard the research vessel “Papanin” at 9 stations of South Baikal and 8 stations of Irkutsk Reservoir, including bays (see Figure 1, Table 1).

Water was sampled with a 5 L Niskin bottle (Volta, Russia) from 0.15, 5, 10, 15, 20 and 25 m depths at stations of South Baikal, and from 0.15, 5 and 10 m (at stations 10, 12, 14, 15, 16, 17) or from 0.15 and 5 m depths (at stations 11, 13) in the reservoir due to its shallowness.

Water temperature and pH were measured with a pH-410 field device (Aquilon, Moscow, Russia) directly in the bottle immediately after rising, by opening the upper cap and inserting the probe inside. Values from each horizon were averaged, and the average values were taken for examination at each of the 17 stations.

Then, integrated 1.2 L samples were prepared by combining 200, 400 and 600 mL from each horizon, respectively. Next, 500 mL of integrated samples was frozen for the further measurement of nutrients in vitro.

Next, 20 mL of each integrated sample was precipitated with a syringe on a 13 mm filter with 0.8 μm pores (Whatman Part of GE HealthCare, Chicago, IL, USA) for SEM. Then, the filter was rinsed with 20 mL of 70% ethanol. A filter with test material was dried at room temperature and fastened to SEM stubs with double tape. It was stored at room temperature before arrival at the laboratory.

### 2.3. Hydrochemistry

Mineral forms of biogenic elements were determined after filtration using membrane cellulose acetate filters with 0.45 μm pores (Vladisart, Vladimir, Russia).

The content of biogenic elements was measured with a PE-5400VI spectrophotometer (Russia): nitrate was measured using salicylic sodium, detection limit 0.1 mgL^−1^ [33], silicon in the form of silicomolybdic heteropoly acid, detection limit 0.1 mgL^−1^ [34], phosphate as phosphomolybdenum complex, detection limit 0.010 mgL^−1^ [35].

### 2.4. Investigation of Silica-Scaled Chrysophytes

The filter with the studied material was coated with gold in an SDC 004 vacuum evaporator (SD 004 Balzers, Liechtenstein) and examined using a QUANTA 200 SEM (FEI Company; Hillsboro, OR, USA). The distributions and relative abundances of each species of chrysophytes were ranked by the number of scales observed on the SEM stub as follows: very rare, 2–25 scales in the stub (+); rare, 26–50 (++); common, 51–250 (+++); and abundant, if the number of scales on the stub was >251 (++++). The geographical distribution of species was determined according to the previously described latitudinal and longitudinal groups of distribution of silica-scaled chrysophytes [36,37]. Affiliation with a certain latitudinal group was marked as: P—polyzonal (species found in all climatic zones); A-Bor—arctic–boreal (species found in the Northern Hemisphere in the temperate and/or arctic zones); Bor—boreal (species only found in the boreal zone). Affiliation with a certain longitudinal group was marked as: C—cosmopolitan (species found on all six continents); W—widespread (species absent from one or two continents); R—species with scattered distribution (species rarely found across different latitudes); End—endemic (species restricted to a certain geographical range in a continent). We used the tag ‘unkn’, i.e., with unknown geographical characteristics, for species only identified to the genus level.

### 2.5. Statistical Analysis

Exploratory analyses of community composition were performed using R package vegan v.2.5-6 [38]. Environmental factors were analyzed for collinearity. Pearson correlation coefficients and their *p*-values were computed for each pair of explanatory variables using R packages corrplot [39] and Hmisc [40]. The correlation matrix was visualized with R package rcorr using hierarchical clustering to group variables. Variables were centered and scaled to have zero means and standard deviations of one. This standardized environmental matrix was used for the constrained ordination of the species abundance of silica-scaled chrysophytes using constrained correspondence analysis (CCA). Both forward selection and backward elimination approaches were tested to produce a model.

## 3. Results

### 3.1. Diversity and Distribution of Silica-Scaled Chrysophytes Depending on Water Parameters

**Figure 2:**
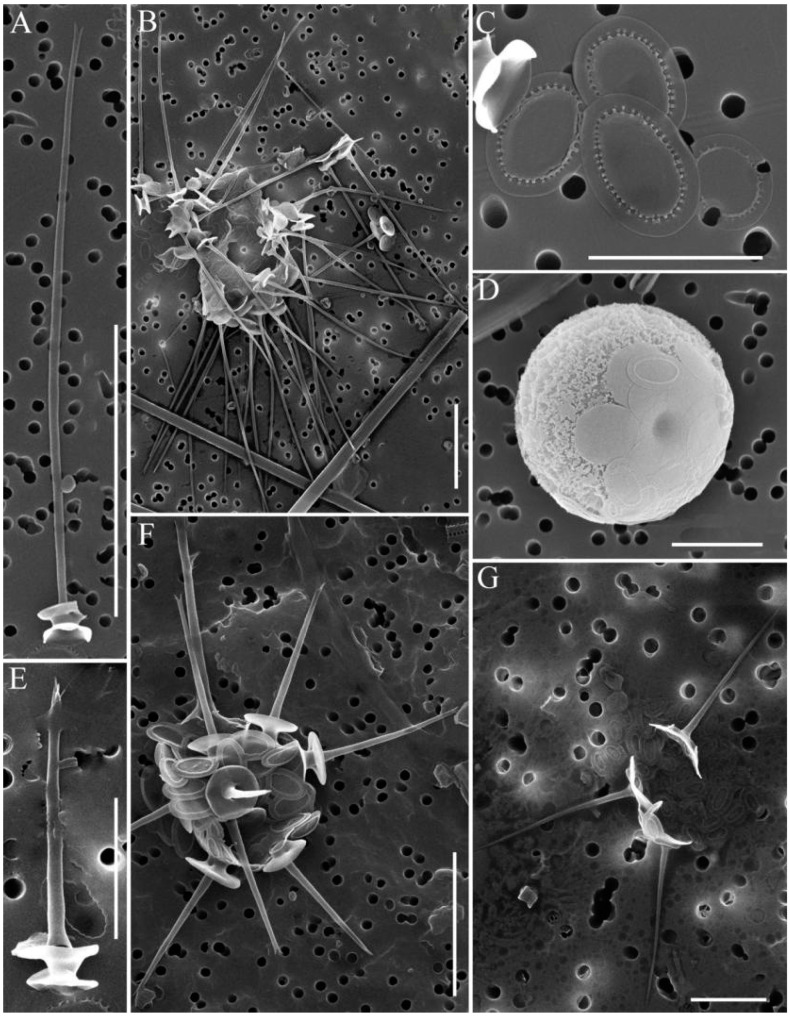
*Chrysosphaerella* taxa from South Baikal and Irkutsk Reservoir, SEM: (**A**–**D**) *Chrysosphaerella baicalensis*, long spine scale (**A**), long spine and plate scales (**B**), plate and spine scales (**C**), forming stomatocyst (**D**); (**E**,**F**) *C. brevispina*, plate scale (**E**), spine and plate scales (**F**); (**G**) *C. coronacircumspina*, spine and plate scales. Scale bars: (**C**–**E**,**G**) 5 μm; (**F**) 10 μm; (**A**,**B**) 20 μm.

**Figure 3:**
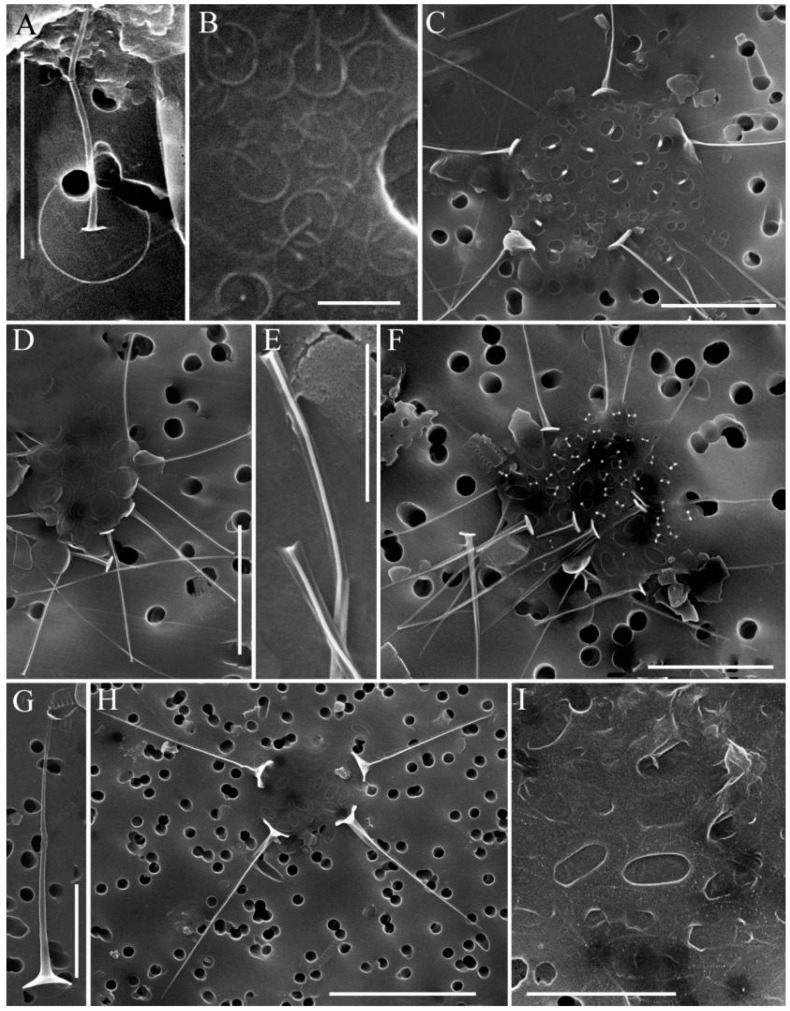
*Spiniferomonas* and *Paraphysomonas* taxa from South Baikal and Irkutsk Reservoir, SEM: (**A**) *Paraphysomonas* sp. 1; (**B**) *P. bandaiensis*; (**C**) *Spiniferomonas triangularis*, spine and plate scales; (**D**,**E**) *S. abrupta*, spine and plate scales (**D**), the tips of the spines (**E**); (**F**) *S. cornuta*, spine and plate scales; (**G**), *S. bourrellyi*, spine scale; (**H**) *S. silverensis*, spine and plate scales; (**H**) *Paraphysomonas* sp. 2, base plate scales. Scale bars: (**B**) 0.5 μm; (**E**) 2 μm; (**A**,**C**,**D**,**F**,**G**,**I**) 5 μm; (**H**) 10 μm.

**Figure 4:**
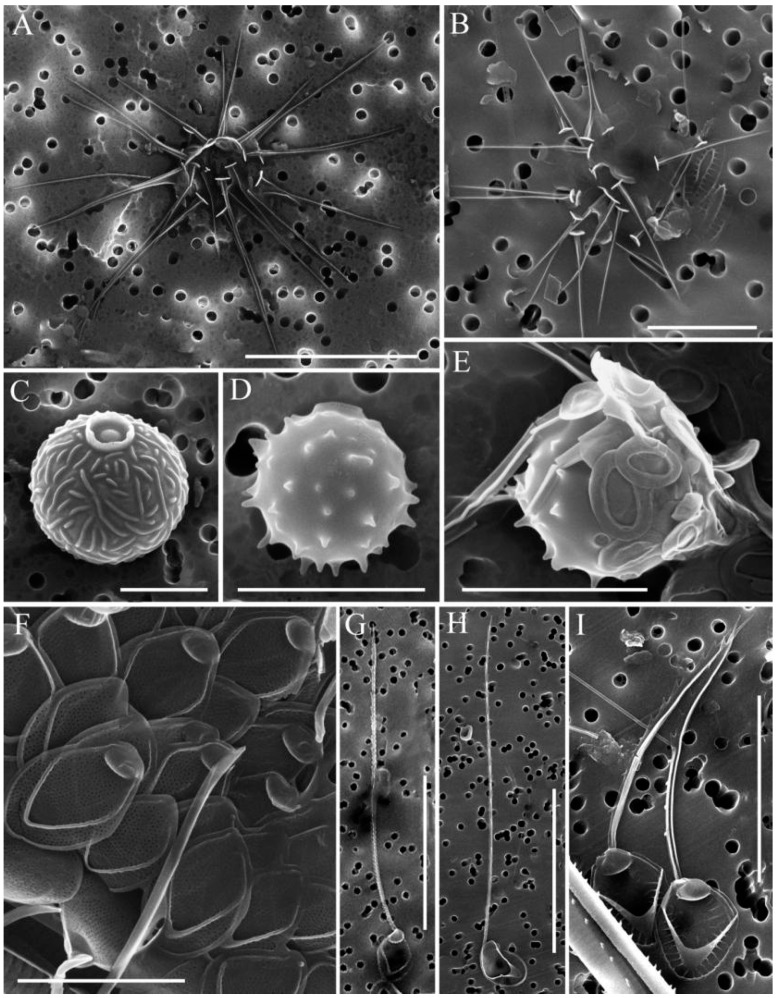
*Spiniferomonas* and *Mallomonas* taxa from South Baikal and Irkutsk Reservoir, SEM: (**A**) *Spiniferomonas trioralis* f. *cuspidata*, long spines and plate scales; (**B**) *S. trioralis*, spines and plate scales; (**C**–**E**) stomatocysts *S. trioralis*, stomatocyst 111, Zeeb et al., 1990 (**C**), stomatocyst 450 Firsova & Bessudova, 2017 (**D**,**E**); (**F**,**G**) *Mallomonas alpina*, scales (**F**), a scale with a long bristle (**G**); (**H**) *M. vannigera*, a scale with a long bristle; (**I**) *M. acaroides*. Scale bars: (**B**–**F**) 5μm; (**I**) 10 μm; (**A**,**G**,**H**) 10 μm.

**Figure 5:**
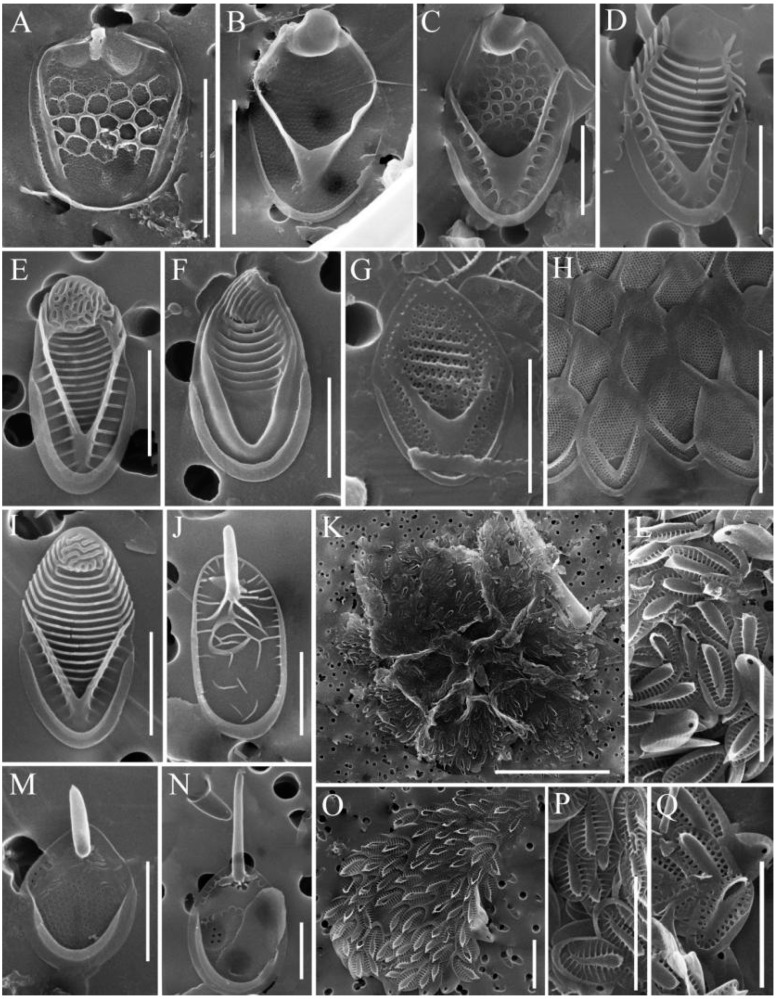
Mallomonas and Synura taxa from South Baikal and Irkutsk Reservoir, SEM: (**A**) Mallomonas punctifera; (**B**) M. elongata; (**C**) M. crassisquama; (**D**) M. striata; (**E**) M. striata var. getseniae; (**F**) M. grachevii; (**G**) M. trummensis; (**H**) M. tonsurata; (**I**) Mallomonas sp. 1; (**J**) Synura punctulosa; (**K**,**O**,**P**) S. cf. glabra; (**L**) Synura sp. 1; (**M**) Synura cf. echinulata; (**N**) S. spinosa f. longispina; (**Q**) Synura sp. 2. Scale bars: (**A**,**C**–**G**,**I**,**J**,**M**,**N**) 2 μm; (**A**,**B**,**H**,**L**,**O**–**Q**) 5 μm; (**K**) 20 μm.

In total, 31 species of silica-scaled chrysophytes were found in the study area: *Chrysosphaerella* (*n* = 3), *Paraphysomonas* (*n* = 2), *Spiniferomonas* (*n* = 8), *Mallomonas* (*n* = 12) and *Synura* (*n* = 6) (Table 2, Figure 2, Figure 3, Figure 4 and Figure 5).

**Figure 6:**
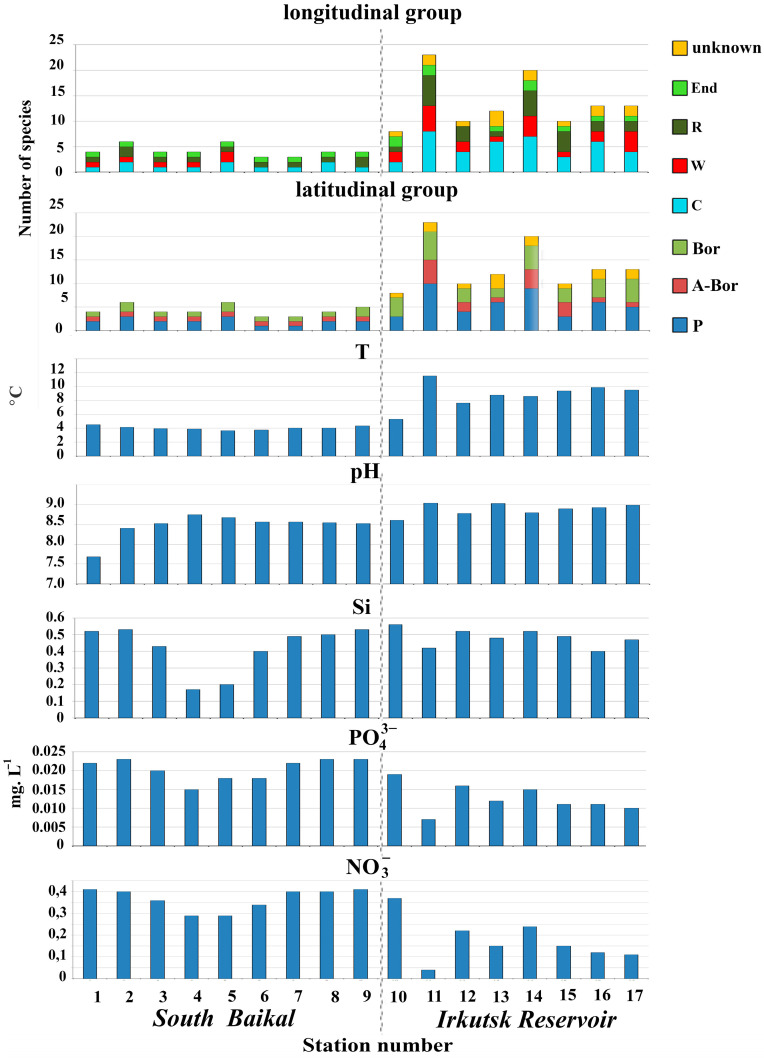
Habitat parameters, distribution of silica-scaled chrysophytes in South Baikal and in Irkutsk Reservoir in June 2023, geographical characteristics of discovered species (C—cosmopolitan; W—widespread; R—rarely found; P—polyzonal; A-Bor—arctic–boreal; Bor—boreal; End—endemic; unknown—possibly new species.

A trend toward increasing temperature and pH alongside decreasing concentrations of nutrients was observed downstream from the lake toward the reservoir (see Table 1, Figure 6).

An increase in the biodiversity of silica-scaled chrysophytes takes place against the following background: only 7 species were found in South Baikal during the study, while 31 species were in Irkutsk Reservoir.

The lowest species diversity was recorded in South Baikal (3–6 species) at T = 3.6–4.5 °C and pH = 7.18–8.24. Hydrochemical parameters at the first station of Irkutsk Reservoir (St. 10, Burduguz) were close to those of South Baikal, except water temperature, which increased at that station up to 5.3 °C. Further to the reservoir (St. 11, Kurma Bay), water temperature and pH achieved their maximal values, 11.55 °C and 8.55, respectively. The species diversity of silica-scaled chrysophytes in Kurma Bay was the highest (23 species) among the stations studied (see Table 1 and Table 2). Further, in the central point at the exit from Kurma Bay (St. 12), the diversity of silica-scaled chrysophytes decreased up to 10 species at water temperature of 7.66 °C and pH of 8.27.

There was less diversity in the species composition at the next site (Elovy Bay; St. 13), situated downstream. Meanwhile, the species diversity increased again up to 20 species in the central point of the reservoir at the exit from Elovy Bay, with water temperature and pH recorded as being 8.63 and 8.29, respectively.

Water temperature and pH values at St. 15–17 located downstream after the central point of the reservoir (St. 14) were again similar (9.4–9.9 °C and 8.39–8.48, respectively); however, the diversity of chrysophytes fell down to 10–13 species (see Table 2).

The species composition of silica-scaled chrysophytes of South Baikal included four typical core representatives of under-ice (March) and spring plankton such as *Chrysosphaerella baicalensis* (endemic of Lake Baikal), *C. brevispina*, *Mallomonas alpina* and *M. vannigera*, as well as three species, *Spiniferomonas trioralis*, *S. trioralis* f. *cuspidata* and *S. bourrellyi*, usually typical of summer–autumn plankton. *C. baicalensis*, *S. trioralis* f. *cuspidata* and sometimes *M. alpina* often occurred in samples from South Baikal, both as individual scales and single cells. Nascent stomatocysts of *C. baicalensis* and mature stomatocysts of *S. trioralis* f. *cuspidata* were also found in the samples. Other species only occurred as individual scales (see Table 2).

At St. 10 (Burduguz) situated towards Irkutsk Reservoir, on the one hand, the same species composition of silica-scaled chrysophytes as in South Baikal was observed, including single scales of *Mallomonas grachevii*, species growing under ice (March) recently described in Lake Baikal. On the other hand, the species composition of silica-scaled chrysophytes was enriched in a thermophilic species *S.* cf. *glabra* and *Synura* sp. 2 new for Lake Baikal.

The most diverse was the species composition in Kurma Bay (St. 11), the largest bay of the reservoir. Exactly starting from this station, a new core of species of silica-scale chrysophytes occurring downstream up to the upstream side of the dam as intact cells is being formed. Intact cells and individual scales of *M. alpina,* colonies and individual scales of *S.* cf. *glabra* were observed. At the same time, *C. baicalensis*, *M. vannigera* and *C. brevispina* species typical of South Baikal remained in the species composition; however, they only occurred as individual scales and spines. Single scales of *M. grachevii* from Lake Baikal were also found there. Species such as *M. alpina*, *S. trioralis*, *S. trioralis* f. *cuspidata* and *S. bourrellyi*, which had finished their growth in South Baikal, still occurred in the bay as intact cells. Single scales of species absent from the lake were found in the bay, but all of them, except *Synura spinosa* f. *longispina*, *Paraphysomonas bandaiensis* and *Mallomonas* sp., are typical of the Baikal region. There are single findings of *Spiniferomonas abrupta* and *S. silverensis* cells, typical of summer–autumn plankton of Lake Baikal.

The species composition dramatically decreased at the central point close to Kurma Bay (St. 12) and in Elovy Bay (St. 13), mainly thanks to the evanescence of the *Mallomonas* species.

At the central point of the reservoir in front of Elovy Bay (St. 14), the species composition was enriched with scales of species that had been found in Kurma Bay. Species of *M. striata*, *M. tonsurata* and *Mallomonas* sp. which had not occurred upstream also enriched the species composition (see Table 2).

When moving towards the upstream side of the dam (St. 17), the species diversity decreased both at the central point, opposite the Ershovsky Bay (St. 15), and in the Ershovsky Bay itself (St. 16). However, the abundance of core-forming species of silica-scaled chrysophytes was unaffected.

**Figure 7:**
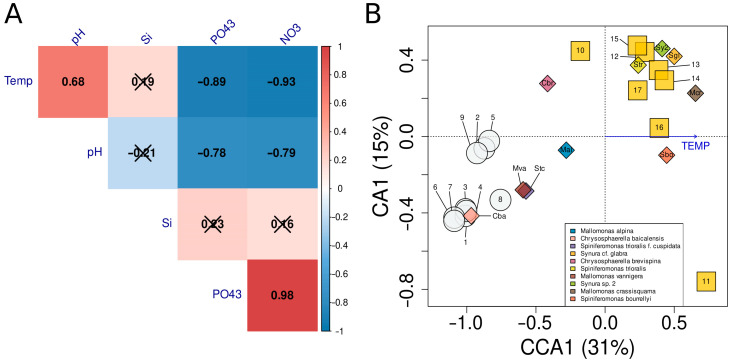
Exploratory analysis of species abundance. (**A**)—Analysis of correlation of environmental parameters. Numerical values are Pearson correlation coefficients with the color legend on the right. Strikeout cells are non-significant correlations (*p* > 0.05). (**B**)—Constrained ordination of the silica-scaled chrysophytes community profiles using correspondence analysis. Gray circles—sampling sites in the south basin of Lake Baikal. Yellow squares—sampling sites across the Irkutsk Reservoir. Diamonds—dominant silica-scaled chrysophytes species. Blue arrow—explanatory variable, used in the model.

Importantly, phosphate and nitrate anion concentrations have strong positive correlation, while both anions negatively correlate with water temperature and pH (Figure 7A). The constrained ordination via CCA generated a model with the single explanatory parameter “temperature” using both forward selection and backward elimination approaches of choosing variables. In this model, temperature alone explained 27% out of 31% of the adjusted total constrained variation in the species abundance matrix (Figure 7B). According to ordination, the silica-scaled chrysophytes community profiles can be split into two groups by sampling location: those from Lake Baikal are different from Irkutsk Reservoir.

### 3.2. Species with Specific Morphology and Undetermined Species

Species suspected to be new and species with peculiarities in morphology were discovered in the study area.

*Paraphysomonas* sp. 1 (Figure 3A). A round base-plate 2.5–2.7 µm in diameter with a dense margin. The central part of the base plate has a looser area. The spine is straight, above 5.5 µm.

*Paraphysomonas* sp. 2 (Figure 3I). The only base plate scale without spines. Elliptical base plate scale (2.7–4.2 × 1.7–2.3 µm). The inner edge of the base plate is ornamented with rows and marginal pegs on the distal side. We had met such scales in the Olenyok River, Yakutia. However, those scales were far smaller (1.6–1.8 × 1.4–1.5 µm) (Figure S2d in [41]).

*Mallomonas* sp. 1 (Figure 5I). Scales are 4.1–4.9 × 2.0–2.3 μm, oval with lateral incurvings. The dome is subcircular with labyrinth-like reticulation. The shield is patterned with 13–15 regularly spaced transverse ribs. The anterior flanges have 8–9 closely spaced struts on each side. The anterior submarginal ribs are well-developed. The V-rib on the scales is acutely angled, hooded. There are pores in the angle of the V-rib above the hood. The posterior rim is wide and smooth. The posterior flange contains approximately 19–20 struts. Bristles were not observed.

Several morphotypes of the *Synura petersenii* sensu lato species complex were observed. We provide descriptions of morphotypes observed in our samples.

*Synura* sp. 1 (Figure 5L). Body scales are 3.1–4.4 × 1.2–2.3 µm. A wide, cylindrical keel ends in a prominent acute tip. The foramen pore on the base plate is circular, 0.26–0.28 mm in diameter. Numerous struts (23–30) extend from the base of the keel towards the margin; sometimes, struts are somewhat reduced, without transverse folds. The rim of the base plate is broad (up to 0.58 µm wide) and encircles more than half the perimeter of scale.

*Synura* sp. 2 (Figure 5Q). Body scales are 3.4–4.2 × 1.7–2.1 µm. The keel is cylindrical, widened anteriorly, and ends in a prominent acute tip. The foramen pore on the base plate is circular, 0.15–0.19 mm in diameter. Numerous struts (23–30) regularly extend from the keel to the edge of the scales and are interconnected by transverse folds. The rim of the base plate is broad (up to 0.48 µm wide) and encircles more than half the perimeter of scale.

A species similar to *Chrysosphaerella brevispina*, but with a series of peculiarities inhabits Lake Baikal. This is *C. baicalensis*. We guess that both these species occur in the lake. *C. baicalensis* (Figure 2A–C) shares with *C. brevispina* the structure of scales and spines. However, the spines of *C. baicalensis* are longer, up to 47 μm. In addition, the spine is cylindrical along the whole length, slightly tapering towards a bifurcate tip. The basement of *C. brevispina’s* spine is a little dilated with regard to the rest of the central part and the top of the spine. The length of *C. brevispina*’s spine in Lake Baikal varies between 7 and 16 μm.

It is noteworthy that several species of silica-scaled chrysophytes inhabiting Lake Baikal have spines and bristles longer than those of the same species from other habitats. At the same time, scales of the “Baikal” species have sizes similar to those of the same species from other habitats. Thus, we observed bristles of *M. alpina* (Figure 4G) up to 37 µm long, though they were previously known to vary between 5 and 35 µm [39,40]. The bristles of *M. vannigera* (Figure 4H) reach 43 µm, being previously known to vary between 14.6 and 41.2 µm [42,43].

## 4. Discussion

### 4.1. Change in Species Composition and Diversity of Silica-Scaled Chrysophytes from Lake Baikal to Irkutsk Reservoir

The south part of the lake lacks large tributaries, unlike the central and northern parts; therefore, a transfer of silica-scaled chrysophytes from tributaries to the pelagic zone of the lake is minimal. This may be a probable reason for the low species diversity of silica-scaled chrysophytes in South Baikal during spring, when no more than 11 species occur [44], while during “low production” years, their number may not exceed 3–6 species according to our data. This trend supports the claim that the species diversity of silica-scaled chrysophytes in oligotrophic waters is low and may consist of only a few species [11,45]. Nevertheless, in June, Irkutsk Reservoir was also oligotrophic (with some mesotrophic traits) [46] according to its hydrochemical parameters, but it had relatively high species diversity.

During the study, we observed the end of the spring bloom of silica-scaled chrysophytes in South Baikal. It is evidenced by the occurrence of single intact cells and stomatocysts of dominating species *C. baicalensis* and *S. trioralis* f. *cuspidata*, as well as individual scales and bristles of *M. alpina*. The low species diversity of silica-scaled chrysophytes typical of South Baikal included *C. baicalensis*, *C. brevispina*, *M. alpina*, *M. vannigera* and *S. trioralis* f. *cuspidata*, usual for springtime. The intermediate area between the lake and the reservoir, St. 10 (Burduguz), is almost completely under the influence of Lake Baikal. Further, the species composition and core of silica-scaled chrysophytes were being enriched simultaneously with increasing water temperature and pH. The core of silica-scaled chrysophytes in Irkutsk Reservoir included *Mallomonas alpina* and *Synura* cf. *glabra* species, and also, cells of *Spiniferomonas trioralis*, *S. trioralis* f. *cuspidata*, *M. acaroides*, *M. crassisquama* and *Synura* sp. 2 occurred. Representatives of the *Synura* genus had been previously mentioned as actively growing, sometimes producing blooms [17], both in waters of a tropical reservoir [10] and in reservoirs of temperate zones such as the Volga–Kama Cascade in Russia [13] and in Hungary [17]. In addition, dominating species in those reservoirs also included *Mallomonas alpina*, *M. crassisquama* and *M. acaroides* [13,17], frequent in Irkutsk Reservoir. *M. alpina*, whose growth in South Baikal had been over, continued growing when it found itself in warmer water of the reservoir. At the same time, other species of South Baikal occurred in the reservoir as single individual scales (see Table 2); this may characterize them as cold-water species. Diatom algae had been previously demonstrated not to undergo such a dramatic change in composition of dominating species. Species of diatom and green algae dominating in South Baikal continue to dominate in the reservoir too [21].

In the literature, we found only one mention of species of the *Spiniferomonas* genus in waters of a reservoir. Five species of the genus were discovered earlier in Khantai Reservoir. Perhaps their growth is characteristic of northern reservoirs. It is interesting that species of the *Spiniferomonas* genus previously considered as typically autumnal [15,44] grow in vernal plankton of the lake (one species) and the reservoir (seven species). We had already described this trend [19] in samples from lakes Labynkyr and Vorota taken under the ice. Species of the *Spiniferomonas* genus refer to mixotrophs. *Spiniferomonas bourrellyi*, *S. trioralis* and *S. trioralis* f. *cuspidata* species develop both in autumn and under ice, having a wide tolerance to water temperature. Other species of the genus occur mainly in autumn. In springtime, cold waters of Lake Baikal restrain the development of autumnal species of the *Spiniferomonas* genus, but they start growing when they get into in warmer waters of the reservoir comparable to the temperature with those of the lake in autumn. Thus, a phenomenon of “diffuseness” of seasonal patterns in growth of some species [1], including silica-scaled chrysophytes, may be observed in reservoirs due to relatively stable hydrological conditions and higher water temperature.

### 4.2. Distribution of Silica-Scaled Chrysophytes Depending on the Water Parameters

Lake Baikal is the world’s deepest and one of the largest lakes. The content of nutrients in the pelagic zone of Lake Baikal is low, characterizing the lake as oligotrophic [28,47,48,49,50]. Hydrochemical parameters in South Baikal during the study (June) were within multiannual observations [47,48,49]. Low concentrations of nutrients in waters of Irkutsk Reservoir also suggest it has a continued status of an oligotrophic to mesotrophic water body [20,21]. The species diversity in the study area in June was affected mostly by T and pH, having, at the same time, a negative correlation with biogenic elements growing from the lake towards the upstream side of the reservoir (Figure 7A). The highest diversity of silica-scaled chrysophytes (23 species) was recorded in the largest bay of the reservoir, in Kurma Bay, at the highest values of temperature (11.55 °C) and pH (8.57) (see Figure 6).

Temperature was one of the factors differentiating the South Baikal and Irkutsk Reservoir communities. The constrained correspondence analysis yielded a model with temperature being the single variable (Figure 7B), explaining almost all total constrained variation in the species abundance matrix. At the same time, temperature strongly negatively correlated with concentrations of phosphate and nitrate anions (Figure 7A). These results highlight that we cannot determine the causal effects of one explanatory variable to another. However, in our settings, it seems reasonable to suggest that the temperature of water is the main factor influencing the composition of the community profiles.

In summer, water temperature in bays of Irkutsk Reservoir runs up to 15.5–20.6 °C [20], creating potentially favorable conditions for its enrichment in thermophilic species of silica-scaled chrysophytes. Their diversity in Kurma Bay may thus follow on from previous seasons thanks to the broad range of temperatures involving the growth of species with different autecology. Water temperature at the upstream side only runs up to 16 °C [20] in summer, being significantly lower than in bays. Only 13 species were found there in June. Both stagnant near-shore zone and a zone of Baikal running water were described at the middle part of Irkutsk Reservoir [51]. Thus, there may occur areas with lower diversity of silica-scaled chrysophytes in the zone with more intensive current and areas with higher species diversity at stations in stagnant waters. So, for example, 20 species were recorded at the central station in Irkutsk Reservoir in the front of Elovy Bay, St. 14.

Silica-scaled chrysophytes are sensitive to water temperature changes; moreover, even a minor change in hydrochemical parameters in a colony (in the case of *Synura*) induces the disintegration of cells to individual scales and spines/bristles [11]. The distribution of silica-scaled chrysophytes along ecological gradients is uneven; their individual species demonstrate a different tolerance to environmental factors [11,45,52]. E.g., many previous investigations had shown the core-forming species *M. alpina* and *M. axaroides* refer to alkaliphilous taxa, inhabiting mostly at high pH values [11]. The *M. acaroides* species is considered pH-indifferent, able to live in a wide range of values [11,45,53]. At the same time, *M. alpina* and *M. acaroides* had been previously described as inhabitants of eutrophic waters of Northern America [11]. However, this is not uncontroversial. For example, there is a report on the prevalence of these species in a reservoir in Hungary, although N and P indicators in the reservoir met mesotrophic conditions according to the author himself [17]. In addition, *M. alpina* prevails among silica-scaled chrysophytes of the vernal plankton of oligotrophic Lake Baikal. A form of *Synura spinosa* f. *longispina* found in the reservoir had been also mentioned previously as typical of oligotrophic waters [11,54,55].

## 5. Conclusions

During the Lake Baikal–Angara River–Irkutsk Reservoir transition in June 2023, increasing water temperature and pH and decreasing concentrations of nutrients were followed by the increasing diversity of silica-scaled chrysophytes, from 7 to 31 species at the expense of cosmopolitan, widespread, as well as rarely found species, and due to polyzonal, arctic–boreal and boreal species in the latitude group. Unknown, possibly new species were discovered in the reservoir too. The composition of prevailing species also was changing at the transition from the lake to the reservoir; only one species with wide ecological valence, *Mallomonas alpina*, was prevailing both in the lake and the reservoir. This succession of species diversity brings evidence that silica-scaled chrysophytes are sensitive components that must be taken into consideration when monitoring reservoirs, with special attention to vegetative stages of individual species.

## Figures and Tables

**Table 1:** Sampling sites in South Baikal and Irkutsk Reservoir and their environmental parameters in June 2023 (site numbers according to Figure 1).

Station Number	Station Name	Coordinates N/E	Water T, °C	pH	Si, mg·L^−1^	PO_4_^3−^, mg·L^−1^	NO_3_^−^ mg·L^−1^
1.	12 km from Kultuk	51°40.578/103°52.309	4.5	7.18	0.52	0.022	0.41
2.	3 km from Marituy	51°45.546/104°13.222	4.1	7.90	0.53	0.023	0.40
3.	Marituy–Solzan	51°38.710/104°13.715	3.9	8.02	0.43	0.020	0.36
4.	3 km from Solzan	51°31.428/104°14.417	3.9	8.24	0.17	0.015	0.29
5.	cape Tolsty–Snezhnaya River	51°36.402/104°44.147	3.6	8.17	0.20	0.018	0.29
6.	3 km from Tankhoi	51°35.440/105°06.968	3.7	8.06	0.40	0.018	0.34
7.	cape Kadilny–Mishikha	51°46.731/105°22.528	4.0	8.06	0.49	0.022	0.40
8.	Listvyanka–Tankhoi	51°42.262/105°00.720	4.0	8.04	0.50	0.023	0.40
9.	3 km from Listvyanka	51°49.033/104°54.616	4.3	8.02	0.53	0.023	0.41
10.	IrkR_Burduguz	52°04.105/104°59.451	5.3	8.10	0.56	0.019	0.37
11.	IrkR_Kurma Bay	52°06.845/104°45.926	11.5	8.57	0.42	0.007	0.04
12.	IrkR_center against Kurma Bay	52°10.874/104°47.935	7.6	8.27	0.52	0.016	0.22
13.	IrkR_Elovy Bay	52°09.906/104°25.172	8.8	8.52	0.48	0.012	0.15
14.	IrkR_center against Elovy Bay	52°14.548/104°45.243	8.6	8.29	0.52	0.015	0.24
15.	IrkR_center against Ershovsky Bay	52°21.511/104°37.550	9.4	8.39	0.49	0.011	0.15
16.	IrkR_Ershovsky Bay	52°20.851/104°34.439	9.9	8.42	0.40	0.011	0.12
17.	IrkR_head water	52°23.478/104°33.722	9.5	8.48	0.47	0.010	0.11

**Table 2:** List of silica-scaled chrysophytes, their geographical distribution and relative abundance in samples from South Baikal and Irkutsk Reservoir in June 2023 (for station numbers, see Figure 1). “+” indicates the relative abundances of each chrysophytes species by the number of scales.

	Species	Latitudinal Group	Longitudinal Group	1	2	3	4	5	6	7	8	9	10	11	12	13	14	15	16	17
1.	*Chrysosphaerella baicalensis* Popovskaya	End	Bor	+++	+++	+++	+++	+++	+++	+++	++	++	++	+		+	+	+	+	+
2.	*C. brevispina* Korshikov	R	Bor		++			++				++	++	+	++		+	+	+	+
3.	*C. coronacircumspina* Wujek & Kristiansen	C	P											+					+	
4.	*Paraphysomonas bandaiensis* Takahashi	R	A-Bor											+						
5.	*Paraphysomonas* sp. 1	unkn	unkn													+				
6.	*Paraphysomonas* sp. 2	unkn	unkn											+						
7.	*Spiniferomonas abrupta* Nielsen	W	Bor											++	++		++			+
8.	*S. bourrellyi* Takahashi	C	P								+			++		++	++			+
9.	*S. cornuta* Balonov	R	A-Bor														+			
10.	*S. silverensis* Nicholls	R	A-Bor											+	+		++	+		
11.	*S. triangularis* Siver	R	A-Bor											+			+			
12.	*S. trioralis* Takahashi	C	P		+			+					+	++	++	++	++	++	+	++
13.	*S. trioralis* f. *cuspidata* Balonov	R	A-Bor	++	++	++	+++	++	++	++	++	++		++	++	++	++	+	++	++
14.	*Mallomonas acaroides* Perty	C	P											+++		++	+++		++	
15.	*M. alpina* Pascher & Ruttner	C	P	+++	++	+++	+++	+++	++	++	++	++	+	++++	++++	++++	++++	++++	++++	++++
16.	*M. crassisquama* (Asmund) Fott	C	P											+++	++	++	++	++	++	++
17.	*M. elongata* Reverdin	W	P											+						
18.	*M. grachevii* Bessudova	End	Bor										+	+			+			
19.	*M. punctifera* Korshikov	C	P											+	+	+				
20.	*M. striata* var. *getseniae* Voloshko	R	A-Bor															+		
21.	*M. striata* Asmund	C	P														+		+	
22.	*M. tonsurata* Teiling	C	P														+			
23.	*M. trummensis* Cronberg	R	A-Bor											+						
24.	*M. vannigera* Asmund	W	P	+	+	+	+	+					+	+			+			+
25.	*Mallomonas* sp.	unkn	unkn														+		+	
26.	*Synura echinulata* Korshikov	C	P											+						
27.	*S.* cf. *glabra* (Korshikov) Škaloud & Kynclová	W	Bor										++	++++	++++	++++	++++	++++	++++	++++
28.	*S. punctulosa* Balonov	W	Bor											+					+	+
29.	*S. spinosa* f. *longispina* Petersen & Hansen	W	P														+			
30.	*Synura* sp. 1	unkn	unkn													++				++
31.	*Synura* sp. 2	unkn	unkn										++	++	++	++	++	++	++	++
	Total			4	6	4	4	6	3	3	4	4	8	23	10	12	20	10	13	13

## Data Availability

Not applicable.
